# GJB3 promotes pancreatic cancer liver metastasis by enhancing the polarization and survival of neutrophil

**DOI:** 10.3389/fimmu.2022.983116

**Published:** 2022-10-19

**Authors:** Yanmiao Huo, Yaoqi Zhou, Jiahao Zheng, Guangxin Jin, Lingye Tao, Hongfei Yao, Junfeng Zhang, Yongwei Sun, Yingbin Liu, Li-Peng Hu

**Affiliations:** ^1^ State Key Laboratory of Oncogenes and Related Genes, Department of Biliary-Pancreatic Surgery, Shanghai Cancer Institute, Ren Ji Hospital, School of Medicine, Shanghai Jiao Tong University, Shanghai, China; ^2^ Department of Interventional Oncology, Renji Hospital, School of Medicine, Shanghai Jiao Tong University, Shanghai, China

**Keywords:** pancreatic cancer, GJB3 (Cx31) gene, cAMP, neutrophil, liver metastasis

## Abstract

Connexins are membrane expressed proteins, which could assemble into hexamers to transfer metabolites and secondary messengers. However, its roles in pancreatic cancer metastasis remains unknown. In this study, by comparing the gene expression patterns in primary pancreatic cancer patients primary and liver metastasis specimens, we found that Gap Junction Protein Beta 3 (GJB3) significantly increased in Pancreatic ductal adenocarcinoma (PDAC) liver metastasis. Animal experiments verified that GJB3 depletion suppressed the hepatic metastasis of PDAC cancer cells. Further, GJB3 over expression increased the neutrophil infiltration. Mechanistic study revealed that GJB3 form channels between PDAC tumor cells and accumulated neutrophil, which transfer cyclic adenosine monophosphate (cAMP) from cancer to neutrophil cells, which supports the survival and polarization. Taken together, our data suggesting that GJB3 could act as a potential therapeutic target of PDAC liver metastasis.

## Introduction

Pancreatic ductal adenocarcinoma is one of the most devastating malignant tumors, with a 5-years survival rate less than 10% and six-month median survival time (1, 2). The poor survival rate is partly owing to its highly distant metastasis (3). The liver is the major destination organ of the disseminated PDAC tumor cells, over 50% PDAC patients suffered with liver metastasis, which lost the chance of operation treatment and without effective treatment strategies currently (4). Thus, there is an unmet demand for investigating the liver metastasis mechanism of PDAC to pave the road to potential targets.

Connexins are proteins that form homomeric or heteromeric hexamers channels, which named as connexons (5). Connexin family is composed by 21 members, which could be further divide into α and β two subtypes (6). Connexins major act as channels to transport small metabolites such as nucleotide, glutamate and glucose between cells (7). There are many functions of connexins in human pathology: Mutation of the *GJB2*, *GJB3* and *GJB6* could leading to the hearing loss (8), *GJB1* mutations in Schwann cells results in demyelinating diseases (9), *GJA1*mutation in keratinocytes causing in skin disorders (10). As for the connexins roles in cancer biology presents as context-dependent effects. At the beginning, connexins were reported as tumor suppressor. They can suppress the growth of glioma and hepatocarcinoma (11, 12). In recent decades, researchers showed that connexins could enhance the invasion ability in melanoma and chemotherapy resistant in lung cancer (13). However, most of proceeding researches focus on the effects to tumor cells themselves. The function and mechanism of connexin regulating tumor microenvironment remain largely unknown, especially in pancreatic cancer.

In this study, we aimed to dissect the contributions of connexins in PDAC liver metastasis. By assessing the connexin family protein in liver metastasis datasets, we found GJB3 expression significantly increased in PDAC liver metastasis and associated PDAC poor prognosis. Further animal studies showed that GJB3 depletion alleviated the liver metastasis by suppressing the amounts and function of tumor associated neutrophil. Mechanism studies showed that GJB3 could transfer cAMP from metastasis PDAC to the neutrophils, which prompting the polarization and survive, resulting in PDAC immune-escape and liver metastasis progress. In summary, GJB3 constitutes a novel pharmaceutically accessible target for both prevention and treatment in PDAC liver metastasis.

## Material and method

### Cell culture

Human PDAC cell line PANC-1 and murine PDAC cell line KPC1199 were obtained from Shanghai Cancer Institute, Ren Ji Hospital, School of Medicine, Shanghai Jiao Tong University and underwent verification in January 2020 by Shanghai Cancer Institute and regular testing (every 3 months) to ensure no contamination with the Mycoplasma. Cells were cultured in the Dulbecco’s modified Eagle’s medium (DMEM) suggested by American Type Culture Collection (ATCC) protocols supplemented with 10% fetal bovine serum (FBS) and 1% penicillin/streptomycin (P/S) at 37°C in a humidified incubator with 5% CO_2_. Cells were maintained at subconfluence and passaged using 0.05% trypsin/EDTA.

### Mouse models for liver metastasis of PDAC

KPC1199 cells (2 × 10^6^ cells) resuspended in 20 μL PBS were injected into the spleen of C57BL/6N male mice (6-8-week-old) using an insulin syringe under 2.5% isoflurane inhalation anesthesia after surgical exposure of spleen. PBS was used as negative control. Four weeks after injection, the mice were sacrificed, and the liver were dissected, weighed photographed and fixed with 4% paraformaldehyde.

### Flow cytometry

Liver tissue were collected at day 7, 14 and 4 weeks after tumor cells intra-splenic injection. The liver was perfused with Liver perfusion medium (Gibco, #17701-038), dissociated by Liver Digest Medium (Gibco, #17703-034), then filtered by 70-μm strainer. Single cell suspensions were centrifuged at 60 g for 1 min and cells in suspension were collected. To detect the existence of immune cells and platelets in liver metastatic tumor, the single cell suspensions were incubated with a mix of pre-labelled antibodies which contained immune cell and platelet markers for surface staining for 30 min at 4°C. After two washes with PBS, the cell suspensions were treated with BD Cytofix/Cytoperm™ solution (BD, #554722) and wash buffer (BD, #554723). Cells were then stained with Zombie Aqua™ Fixable Viability Kit (Biolegend, #423101). Flow cytometry analyses were carried out on a BD LSR-Fortessa (Becton Dickinson) and FlowJo v.10.4.2 was used for further analysis.

### Hematoxylin and eosin staining

The livers of mice were removed and fixed with 10% formalin to prepare paraffin-embedded tissue sections (5 μm). Eight 5 µm sections spaced 100 µm apart at the largest coronal section were evaluated in each mouse. Hematoxylin and eosin (H&E) staining were conducted and microscopic counting was applied. The average number of nodules on each section was taken as the final number of liver metastatic nodules. The nodules with the largest diameter above and below 500 μm were counted separately. Liver metastatic tumor area on sections was counted with image J. N = 5 mice per group.

### Immunohistochemistry

Immunohistochemistry (IHC) were performed routinely. Immunohistochemical staining was performed on the TMA and paraffin sections as previously described. Briefly, the slices were deparaffinized and rehydrated. The sections were blocked in 10% BSA and then incubated with primary antibodies overnight at 4°C and with HRP conjugated secondary antibodies for 1 hour at room temperature. Then sections were then treated with DAB substrate (Thermo, #S21024-2) and counterstained by hematoxylin.

### Lentivirus production and cell transduction

The cDNA encoding mouse *GJB3*, and shRNA constructs against mice *GJB3* and scrambled sequences were purchased from GeneCopoeia (Shanghai, China). Lentivirus particles were generated using the psPAX2 and pMD2.G packaging system. Lentivirus packaging was performed in 293T cells with Lipofectamine 2000 (Invitrogen, Carlsbad, CA) according to standard protocols. Cells were infected with 1 × 10^6^ recombinant lentivirus-transducing units in the presence of 6 mg/ml polybrene (Sigma-Aldrich, #H9268). Twenty-four hours after infection, cells were treated with 5 μg/ml puromycin (Gibco, #A1113802). The overexpression or knockdown cells were selected in the presence of 5μg/ml puromycin for 5 days for stable cell lines. The puromycin-resistant cells were passaged and the knockdown or overexpression efficiency of *GJB3* was verified by real-time qPCR and western blotting.

### Western blotting

Total protein was collected from cells or tissues in RIPA lysis and extraction buffer (Beyotime, Shanghai, China) with 1 × Omplete™ Protease Inhibitor Cocktail (Roche, #4693116001) and 1× phosphatase inhibitors (Roche, #04906845001). Protein concentration was quantified using a BCA protein assay Kit (Pierce Biotechnology). Proteins in the lysates were separated by sodium dodecyl sulfate polyacrylamide gel electrophoresis (SDS-PAGE) and transferred to nitrocellulose (NC) membranes. Next, membranes were blocked with 5% skimmed milk and incubated overnight at 4°C with one of the following antibodies: GJB3 (1:1000, proteintech, #12880-1-AP) and anti-beta-actin (1:3000, Abways, #AB0035). The next day, the membranes were incubated with species-specifc goat anti-mouse (1:10000, Jackson ImmunoResearch, 115-035-003) or goat anti-rabbit (1:10000, Jackson ImmunoResearch, 111-035-003) secondary antibodies for 1 hour at room temperature. Bands were detected by the Odyssey imaging system (LI-COR Biosciences, Lincoln, NE) using enhanced chemiluminescence (ECL, KeyGen Biotech).

### Quantitative Real-time PCR

Total RNA was extracted from indicated cells using RNAiso Plus (Takara, #9108Q), and the PrimeScript™ RT Master Mix (Takara, RR036A) was used to synthesize cDNAs according to the manufacturer’s instructions. Quantitative real-time PCR analyses were performed with SYBR Premix Ex Taq (Takara, Japan) on a 7500 Real-time qPCR system (Applied Biosystems) at the recommended thermal cycling settings: an initial cycle at 95 °C for 15 min followed by 40 cycles of 15 sec at 94 °C and 30 sec at 60 °C. Relative mRNA expression was calculated by normalizing with the β-actin gene expression according to 2^(−ΔΔCt)^ method. Sequences of specific primers were listed below:*β-actin*:forward sequence GGATTCCATACCCAAGAAGGA; reverse sequence GAAGAGCTATGAGCTGCCTGA; *GJB3*: forward sequence CCTCCTCCTATGGACTGCCC; reverse sequence AAGGCCGTGAAGTCTGGGATA; *β-ACTIN*: forward sequence CATGTACGTTGCTATCCAGGC; reverse sequence CTCCTTAATGTCACGCACGAT;

### CAMP quantification

The cAMP quantification was performed as according to the manufactures’ guidelines Briefly, GJB3 negative control or shGJB3 PDAC cells were seeded into six-well plates and cultured for 48 hours, with triplicate samples for each condition. Changes in cAMP concentration were determined using the cAMP-Glo Assay (Promega), which was further quantified by using the MassHunter package.

### CAMP transfer

For cancer cell-neutrophil transfer, the shNC and shGJB3 knockdown PDAC cell lines were seeded as monolayer, which was further incubated with 2 µM fluo-cAMP (Biolog Life Science Institute) at 37°C for 30 min. cAMP- labeled PDAC cells were washed three times with PBS, and then the neutrophil (recipient) were added into the cell culture plates and incubated for 5 hours, then, the neutrophil was isolated from co-culture, washed with PBS, and cAMP transfer was quantified by measurement of total fluorescence using a Tecan fluorescent plate reader.

### Cell viability assay

Cell viability was measured using a cell counting Kit-8 (CCK-8, Dojindo Molecular Technologies, Japan, #CK04). OV-Vector or OV-*GJB3* KPC1199 cells were seeded into 96-well plates and co-cultured with platelets for 72 hours in the presence of 2% FBS. At day0, 1,2 3, and 4, CCK-8 was added into the plates with shNC or shGJB3 cells and which incubated 1hr, following in OD 450 nm Measurement by using microplate reader (BIO-TEK).

### Statistical analysis

Statistical analysis for each experiment was performed using SPSS 22.0 for Windows (version 22.0, SPSS Inc., Chicago, IL, USA). Unpaired two-tailed independent samples *t*-test and the paired *t*-test were used to estimate statistically significant differences between the two groups, respectively. One-way analysis of variance (ANOVA) followed by two-tailed *post hoc* Tukey’s multiple comparison test was used for multiple comparisons. All data were presented as the means ± SD if not indicated. *P* < 0.05 was considered statistically significant.

## Results

### GJB3 expression upregulated in PDAC liver metastasis and associated with poor prognosis

**Figure 1:**
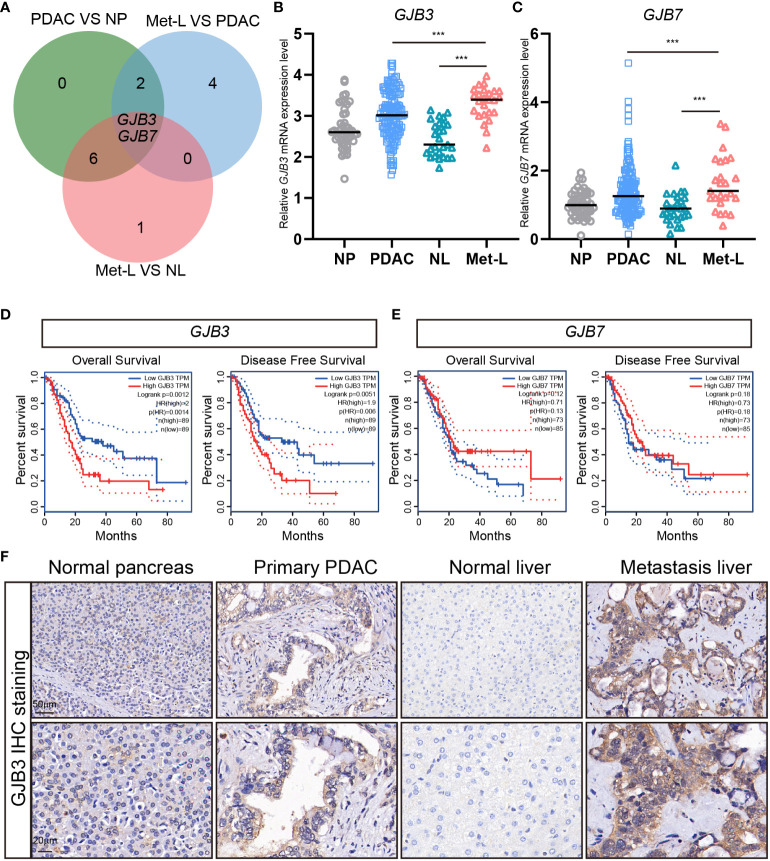
The expression of GJB3 in PDAC liver metastasis. **(A)**. The Veen diagram of connexins genes that increased in primary PDAC and liver metastasis of PDAC. NP, normal pancreas; PDAC, primary pancreatic cancer; NL, normal liver; Met-L, liver metastasis of pancreatic cancer. **(B, C)**. The normalized mRNA expression of *GJB3* and *GJB7* in normal pancreas (n = 46), primary pancreatic cancer (n = 145), normal liver (n = 27) and liver metastasis (n = 25) of pancreatic cancer. **(D, E)** The overall survival and disease-free survival of GJB3 and GJB7 in the TCGA PDAC cohort. **(F)**. The IHC staining of GJB3 in normal pancreas, primary pancreatic cancer, normal liver and liver metastasis of pancreatic cancer. Scale bar is 50 μm for the up panel and 20 μm for the down panel. Data presented as mean ± standard deviation. ***P < 0.001.

To investigating the roles of connexin protein in liver metastasis, we analyzed the expression of total 21 connexins family member in PDAC liver metastasis dataset. We found only two member of b subtype GJB3 and GJB7 mRNA expression are dramatically up-regulated in liver metastasis, when compared to primary PDAC and normal liver tissues (**Figure 1A**). Further, the normalized transcriptome of specimen showed that the based mRNA expression of *GJB3* was much higher than *GJB7* (**Figures 1B, C**). Additionally, the expression changes of GJB3 among liver metastasis and primary PDAC much more obvious than that GJB7 (**Figures 1B, C**). Next, the clinical prognosis association of GJB3 and GJB7 with PDAC was analyzed by Kaplan–Meier. The analysis results showed GJB3 expression significantly associated with overall survival and diseases free survival. Compared to the GJB3 high group patients, GJB3 low group patients extended 10 months’ overall survival time and diseases free survival time (**Figure 1D**). As for GJB7, the patient’s survival time presented no difference among GJB7 high and low expression group (**Figure 1E**). Furthermore, we detected the GJB3 expression by immunohistochemistry (IHC) staining. The IHC results showed GJB3 barely expressed in normal liver tissue only slightly expressed in normal pancreas tissue. In the primary PDAC and liver metastasis, GJB3 exhibited strong expression and presented step by step up-regulation from normal pancreas, primary PDAC to liver metastasis. Together, these results indicated GJB3 play an important role in the progression of PDAC liver metastasis.

### GJB3 depletion suppressed PDAC liver metastasis *in vivo*

**Figure 2:**
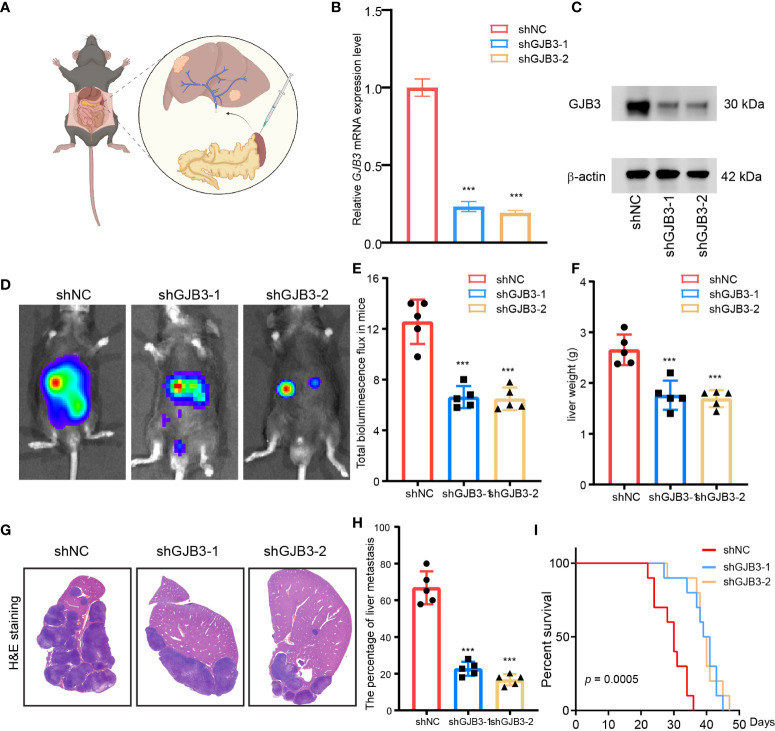
GJB3 depletion suppressed PDAC liver metastasis in mice model **(A)**. Schematic diagram of PDAC mice liver metastasis model. **(B)**. The relative GJB3 mRNA expression level in shNC, shGJB3-1 and shGJB3-2 KPC1199 cells. **(C)**. The immunoblots of GJB3 in shNC, shGJB3-1 and shGJB3-2 KPC1199 cells. **(D)**. The Bioluminescence imaging of shNC, shGJB3-1 and shGJB3-2 KPC1199 cells generated liver metastasis. **(E)**. The total bioluminescence total flux of shNC, shGJB3-1 and shGJB3-2 group mice (n = 5). **(F)**. The measurement of liver weight derived from shNC, shGJB3-1 and shGJB3-2 group mice (n = 5). **(G)**. The Representative H & E staining of liver in shNC, shGJB3-1 and shGJB3-2 group mice. **(H)**. The statistical results of the area invaded by PDAC cells. **(I)**. The overall survival of mice in shNC, shGJB3-1 and shGJB3-2 group (n = 10). Data presented as mean ± standard deviation. ***P < 0.001.

Next, to investigate the function of GJB3 in PDAC liver metastasis, we established mice PDAC liver metastasis by injected Luc expressed PDAC mice cell line KPC1199 into the spleen (**Figure 2A**). We thus established stable GJB3 knockdown cell line by infected with shGJB3 lenti-virus. The knockdown efficiency was analyzed at both mRNA and protein level. The real time PCR and immunoblots results showed GJB3 expression was reduced about 85% by shGJB3 lenti-virus infection (**Figures 2B, C**). Then, shNC and shGJB3 expressed PDAC mice cell lines were used to establish the mice liver metastasis model. Bioluminescence imaging was used to detect the liver metastasis progress. The bioluminescence images indicated that GJB3 depletion greatly reduced the liver metastasis burden in mice (**Figure 2D**). The bioluminescence total flux data showed that shGJB3 cell developed liver metastasis exhibited about 40% reduced (**Figure 2E**), when compared to the shNC group mice. Resected liver measurement showed that weight of liver derived from shGJB3 group mice showed 30% less than negative control group mice (**Figure 2F**). Then, the metastases livers were fixed and sliced, following hematoxylin and eosin (H&E) staining (**Figure 2G**). The metastases liver area was calculated. The statistical results also indicated that GJB3 knockdown alleviate the liver metastasis in mice model. Moreover, the tumor cell growth index was measured by PCNA IHC staining. Consistently, the silencing of GJB3 dramatically suppressed the growth of tumor cell in the liver metastasis (**Supplementary Figure 1**) Further, we compared the overall survival time of shNC and shGJB3 group mice by Kaplan–Meier analysis and found that the median survival time of mice extend form 31 days to 41 days upon GJB3 silenced. In summary, GJB3 knockdown impeded PDAC liver metastasis in mice.

### GJB3 promotes neutrophil accumulation in the PDAC liver metastasis

**Figure 3:**
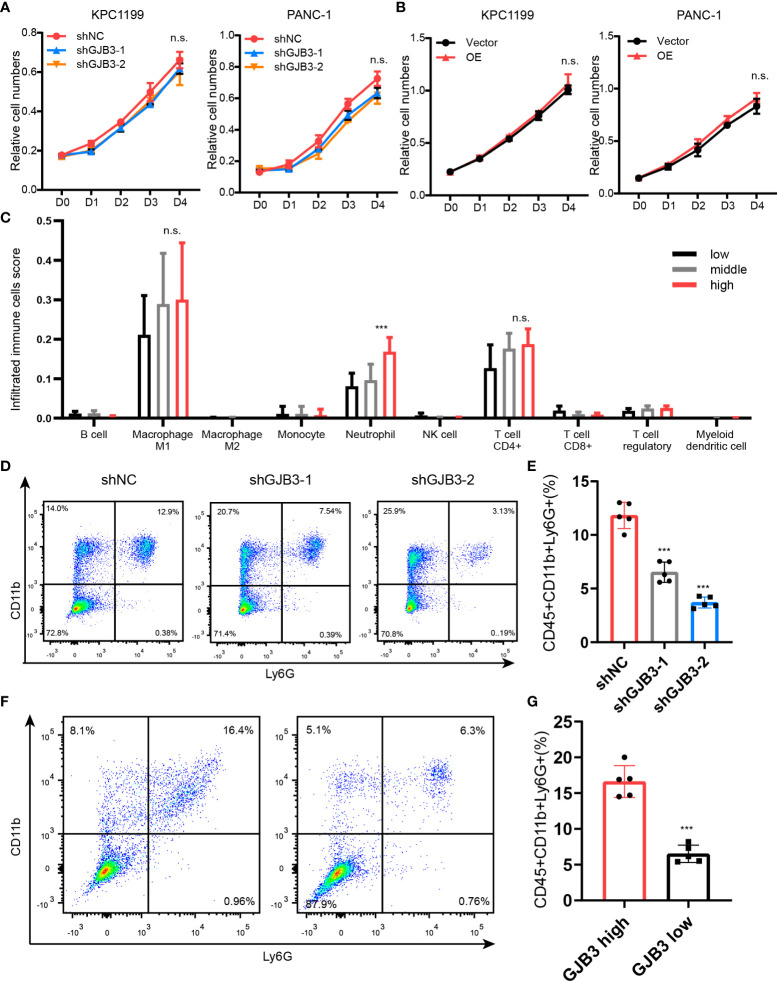
GJB3 knockdown reduced the neutrophil accumulation in liver metastasis. **(A)**. Cell proliferation assay of KPC1199 and PANC-1 cell with GJB3 knockdown or not. **(B)**. Cell proliferation assay of KPC1199 and PANC-1 cell with GJB3 ectopic expression or not. **(C)**. The estimation of immune cells infiltration in the PDAC liver metastasis. **(D, E)**. The flow cytometry of neutrophil infiltrated in the liver from shNC and shGJB3 group mice (n = 5). **(F, G)**. The flow cytometry of neutrophil infiltrated in the GJB3 high and low expression PDAC liver metastasis specimens (n = 5). Data presented as mean ± standard deviation. ***P < 0.001. ns, no significance.

Next, to gain insights into the mechanism of how GJB3 regulate PDAC liver metastasis. We firstly evaluated whether the GJB3 could directly affects the proliferation of PDAC tumor cell. Thus, we measured the cell proliferation of shNC, shGJB3-1 and shGJB3-2 cells. However, the cell viability assay showed that there is on difference between control and shGJB3 cells (**Figure 3A**). To further confirm, the GJB3 was depleted in human PDAC cell lines. In line with mice PDAC cell. GJB3 depletion did not affect the growth of human PDAC cell. (**Figure 3A**). Moreover, we ectopic expressed GJB3 in both mice and human PDAC cells. Consistently, GJB3 over-expression presented no effect on tumor cell growth. (**Figure 3B**). Next, we postulate whether GJB3 could regulate the immune tumor microenvironment. To this end, the liver specimens was dived into three group according to the GJB3 expression as high, middle and low group. We analyzed the immune cell infiltration by using immune deconvolution methods (14). The estimated score showed that macrophage neutrophil and CD4+ T cell were the major three group cells (**Figure 3C**). Among these three groups only neutrophils present significantly difference between GJB3 high and low liver metastasis tissues. To confirmed this, we dissected the liver tissues derived from liver metastasis, flow cytometry data showed that the mounts of neutrophil significantly decreased (**Figures 3D, E**). To further confirm this in human liver tissue, GJB3 high and low expression PDAC liver metastasis specimens were collected and analyzed by flow cytometry. Similarly, the percentage of tumor associated neutrophils in GJB3 high is about three times compared to the GJB3 low expression counterpart (**Figures 3F, G)**. Taken together, these results suggested GJB3 promotes liver metastasis by regulating the neutrophils in the PDAC liver metastasis microenvironment.

### GJB3 facilities neutrophil polarization and survival

**Figure 4:**
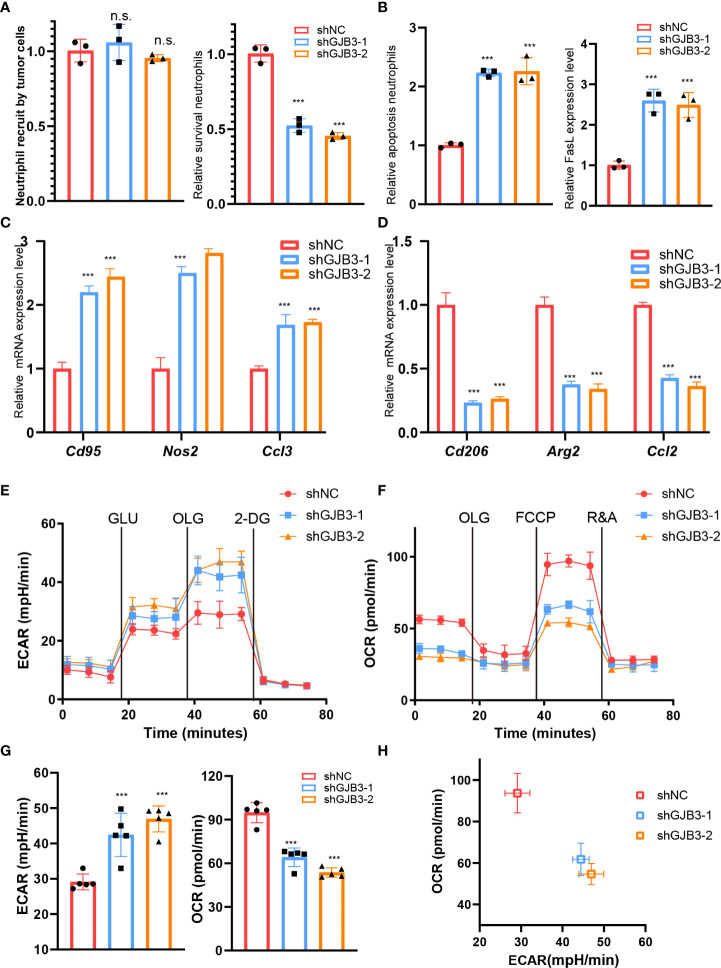
GJB3 facilities neutrophil polarization and survival **(A)**. The relative *Cd95*, *Nos2* and *Ccl3* mRNA expression level in neutrophil which co-cultured with shNC, shGJB3-1 and shGJB3-2 KPC1199 cells. N1- like maker genes. **(B)**. The relative *Cd206, Arg2* and *Ccl2* mRNA expression level in neutrophil which co-cultured with shNC, shGJB3-1 and shGJB3-2 KPC1199 cells. **(C–E)**. The extracellular acid rate measurement and oxygen consumption rate measurement of neutrophil which co-cultured with shNC, shGJB3-1 and shGJB3-2 KPC1199 cells (n = 5). **(F)**. The metabolism phenotype of neutrophil which co-cultured with shNC, shGJB3-1 and shGJB3-2 KPC1199 cells. **(G)**. The neutrophil recruitment by shNC, shGJB3-1 and shGJB3-2 KPC1199 cells (n = 3). **(H, I)**. The survival and apoptosis neutrophil upon co-cultured with PDAC cells (n = 3). **(J)**. The relative FasL expression level in neutrophil. Data presented as mean ± standard deviation. ***P < 0.001. ns, no significance.

Given that the amount of neutrophil increased, we, thus, evaluated whether GJB3 could affect the recruitment and survival of neutrophils. The results showed GJB3 knockdown barely influence the recruitments of tumor cells to neutrophil (**Figure 4A**), but greatly reduced the survival of neutrophil (**Figure 4A**). To further confirm this, we measured the apoptosis cell and death inducing signaling complex associated genes FasL expression. As expected, GJB3 depletion promotes the neutrophil apoptosis and FasL expression (**Figure 4B**). To further characterize the influence of GJB3 on neutrophil, the mice neutrophil was isolated and co-cultured shNC or shGJB3 mice PDAC cells. Given that plasticity of neutrophils, the typical N1 and N2 neutrophil maker genes expression were detected in neutrophils that co-cultured with control or shGJB3 KPC1199 cells. The real-time PCR indicated that N1 related genes *Cd95*, *Nos2* and *Ccl3* presented greatly up-regulation in neutrophils that cultured with shGJB3-1 and shGJB3-2 mice PDAC cells (**Figure 4C**). As for N2 genes *Cd206, Arg2* and *Ccl2* exhibited more than 60% reduction upon GJB3 silenced (**Figure 4D**). Considering that the metabolism greatly involved into the function of immune cells, thus, both the glycolysis and mitochondrial oxidative phosphorylation were measured by seahorse assay. Consistently, extracellular acid rate measurement showed the glycolysis ability significantly enhanced upon GJB3 knockdown (**Figure 4E**). On the contrary, the oxygen consumption rate dramatically reduced when cultured with GJB3 depleted mice PDAC cells (**Figures 4F, G**). Further, metabolic phenotype analysis showed the metabolism of neutrophil switched from mitochondrial oxidative phosphorylation to glycolysis (**Figure 4H**). These data indicated that the immune suppression ability was impaired, and the anti-tumor ability was strengthened. In summary, the GJB3 facilities neutrophil polarization and survival in the PDAC liver metastasis.

### GJB3 transfer cAMP to regulating neutrophil polarization and survival

**Figure 5:**
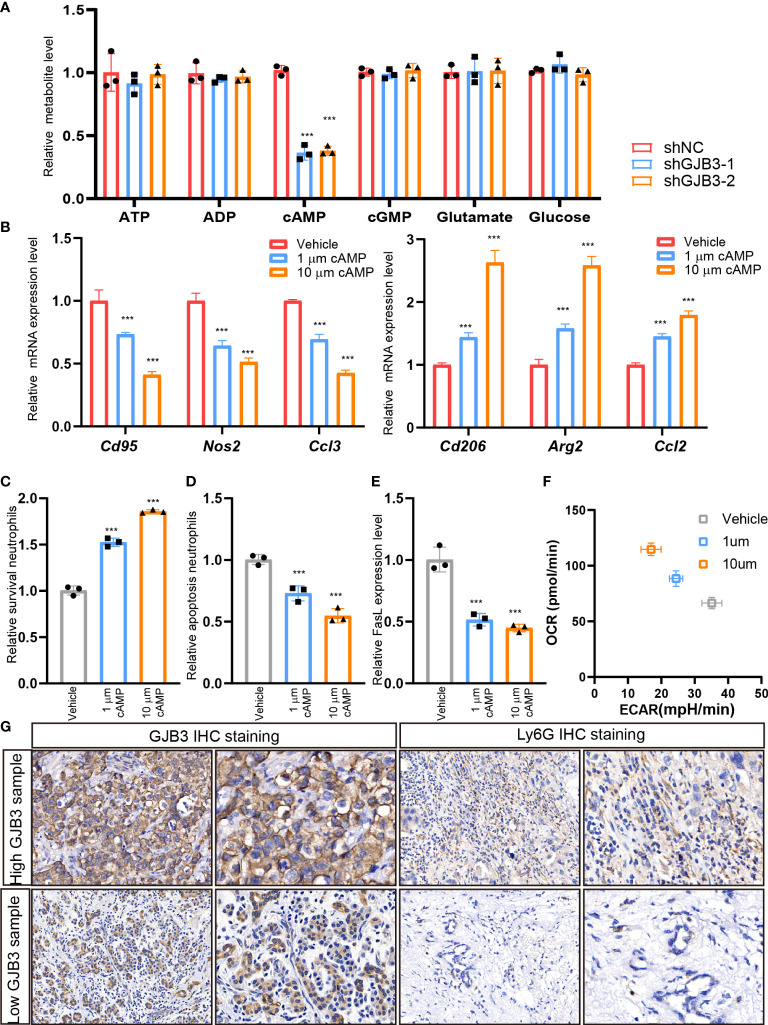
GJB3 transfer cAMP to regulating neutrophil polarization and survival **(A)**. The quantification of metabolites in the culture supernatant from shNC, shGJB3-1 and shGJB3-2 KPC1199 cells. **(B)**. The relative *Cd95*, *Nos2*, *Ccl3*, *Cd206, Arg2* and *Ccl2* mRNA expression under KPC cells treated with 1μm cAMP, 10μm cAMP. **(C, D)**. The survival and apoptosis neutrophil upon treated with different dose of cAMP. **(G)**. The IHC staining of GJB3 and Ly6G in human liver metastasis specimens. Scale bar is 50 μm. Data presented as mean ± standard deviation. ***P < 0.001.

Next, we aimed to investigate the mechanism of GJB3 regulate neutrophil. As GJB3 could form a channel to transfer metabolites to outside of cells. Thus, we detected the level of nucleotide, glutamate and glucose upon GJB3 silencing. We found that the extracellular cAMP level significantly reduced, and other metabolites barely altered (**Figure 5A**). Conversely, ectopic GJB3 expression significantly increased the extracellular cAMP level (**Supplementary Figure 2A**). These results indicated that cAMP potential mediated the neutrophil polarization and survival. To confirm this hypothesis, we treated neutrophil with different doses of cAMP and detected the N1/N2 maker genes expression. As expected, cAMP significantly repressed the N1 related genes expression and the induced N2 related genes expression (**Figure 5B**). Next, we evaluated the neutrophil survival upon treated with cAMP. The ratio of survival neutrophil increased presented as dose-dependent manner (**Figure 5C**). In line with this, the apoptosis cell and death inducing signaling complex associated genes *FasL* expression significantly suppressed by cAMP (**Figures 5D, E**). Also, cAMP treatment triggered a metabolic shift from glycolysis to mitochondrial oxidative phosphorylation (**Figure 5F**). To further confirm this, we collected GJB3 high and low expression liver metastasis tissue and measured the cAMP level in metastasis microenvironment. And indeed, the cAMP level in strong GJB3 expression liver metastasis tissues much higher than that in the weak GJB3 expression liver metastasis tissues (**Supplementary Figure 2B**), also more neutrophil infiltration (**Figure 5G**). Taken together, GJB3 could transfer cAMP into the neutrophil to enhance the neutrophil survival and immune suppression polarization, finally promoted the liver metastasis of PDAC.

## Discussion

Currently, PDAC liver metastasis gradually become the deadliest threaten for the prognosis and survival (15, 16), which could not be cured by surgery resection or chemotherapy. Recently, reshaping the immunosuppressive environment attracted lots of attention due to the successful applied of immune checkpoint blockage such as programmed death ligand-1 (PD-L1) and cytotoxic T-lymphocyte associated protein 4 (CTLA-4) for therapizing multiple types of tumors (17). Nowadays, extensive evidence indicated that neutrophils play crucial roles in tumor metastases, especially for liver and lung metastasis in gastroenterology cancers (18, 19). However, how metastasis PDAC tumor cell regulates neutrophils has not been fully understanding. In this study, we found that GJB3 expressed tumor cells could transfer cAMP into the neutrophils, which promoting the metabolism reprograming, N1 to N2 switch and survive, which finally promotes the tumor cells immune escape and PDAC liver metastasis progress.

To date, the roles of connexins in tumorigenesis have been researched for more than 50 years (20). The proceeding studies reported the complexity functions of complexity connexins during the development of cancer. At the beginning, connexin protein such as Cx43 and Cx32 recognized as tumor suppressor gene, which could inhibit the embryo cells and glioma proliferation (21, 22). After 2000, evidence for connexin also could act as oncogenes gradually presented. Extensive studies showed that some connexin proteins such as Cx43 expression up-regulated in the brain metastases of breast cancer (23), Cx26 greatly expressed in the late-stage tumors (24), and Cx40 could regulate the angiogenesis to promote cancer metastasis (25). Consist in this, by analyzing the publicized GEO datasets and IHC analyzing, we found GJB3 (Cx31) expression significantly increased in the liver metastasis, when compared to the primary PDAC tissue, and high GJB3 expression associated with poor prognosis. Similarly, Yuanlin et al. reported high GJB3 expression associated with an unfavorable prognostic performance (26). On the contrary, Deqiang et al. reported that Cx31.1 could suppress non-small cell lung cancer metastasis by impeding the switch of tumor cell from mesenchymal towards to epithelial phenotype (27), which further confirmed that the connexin protein functions and roles varies in different cancer types. As for the roles of GJB3 in other cancer metastasis need further researches.

Extensive studies reported that neutrophils into the metastasis tumor microenvironment to promote tumor distant metastasis (28). Neutrophils could release immunomodulators, chemokines and complement to enhance the tumor cell colonization and immune escape, which were greatly regulated by the metastasis’s tumor cells and local resident stromal cells. Tumor cell released granulocyte-macrophage colony stimulating factor to educate the neutrophil, which promoted the angiogenesis of tumor microenvironment (29). Recently, Zhiyuan et al. reported that lung mesenchymal stromal cells produced complement 3 (C3) could recruit the neutrophil and enhanced the formation of neutrophil extracellular traps (30). In our work, we found that tumor cell derived metabolites could also be regulating the neutrophil functions. We found high GJB3 expressed PDAC tumor cell could transport their cAMP to the neutrophil in the liver metastasis tumor microenvironments, which promote the survival and polarization. Consist with our studies, proceeding reports also showed that extracellular metabolites could play a crucial role in tumor progression. Pancreatic stellate cell was found could release non-essential amino acids to fuel the growth of PDAC cells (31). Colorectal cancer cell could uptake the extracellular phosphocreatine to satisfy the highly needs of energy (32). In our projects, the cAMP could promote the survival of neutrophil. In line with this, Kinsey reported that leukotriene B4 receptor activation on neutrophil could elevated the cAMP level, which suppressed the death-inducing signaling complex formation, which reduced the apoptosis of neutrophil (33). Also, cAMP was reported as a potent inhibitor of effector tumor-specific T cells (34). Furthermore, cAMP is involved in Treg-mediated suppression, resulting in suppressing interleukin (IL)-2 production and subsequent CD4+ T-cell proliferation (35). Recent studies have demonstrated that high concentrations of cAMP exist in tumor cells and tumor-derived endogenous cAMP is responsible for the induction of T-cell senescence (36, 37). Collectively, these researches indicated that targeting cAMP could be a potential strategy to remodeling the tumor immune microenvironment. Our data provided a new insight into the regulation of neutrophils by cancer cell in liver metastasis microenvironment by exhibited the new way of cAMP to the neutrophil metabolism and survival.

In summary, our findings delineated that GJB3 could promotes pancreatic cancer liver metastasis by enhancing the polarization and survival of neutrophil, which could be sever as a potential target for PDAC liver metastasis

## Data availability statement

The raw data supporting the conclusions of this article will be made available by the authors, without undue reservation.

## Ethics statement

The studies involving human participants were reviewed and approved by Ren Ji Hospital, School of Medicine, Shanghai Jiao Tong University. The patients/participants provided their written informed consent to participate in this study. The animal study was reviewed and approved by Institutional Animal Care and Use Committee (IACUC) of Shanghai Jiao Tong University.

## Author contributions

Conceptualization, YH, YS, YL, and L-PH; Methodology, YZ, JHZ, GJ, LT, and HY; investigation, YH and YZ; formal analysis, YH, YS, and L-PH; writing, YH and L-PH; supervision, JZ, YS, YL, and L-PH. All authors contributed to the article and approved the submitted version.

## Conflict of interest

The authors declare that the research was conducted in the absence of any commercial or financial relationships that could be construed as a potential conflict of interest.

## Publisher’s note

All claims expressed in this article are solely those of the authors and do not necessarily represent those of their affiliated organizations, or those of the publisher, the editors and the reviewers. Any product that may be evaluated in this article, or claim that may be made by its manufacturer, is not guaranteed or endorsed by the publisher.
