# Supplementary material for: GJB3 promotes pancreatic cancer liver metastasis by enhancing the polarization and survival of neutrophil
Source: Front Immunol. 2022 Oct 19;13:983116. doi: 10.3389/fimmu.2022.983116 (PMC9627207; doi:10.3389/fimmu.2022.983116)
Supplement: Supplementary file 1 [file DataSheet_1.docx]

Supplementary figure

Supplementary figure 1


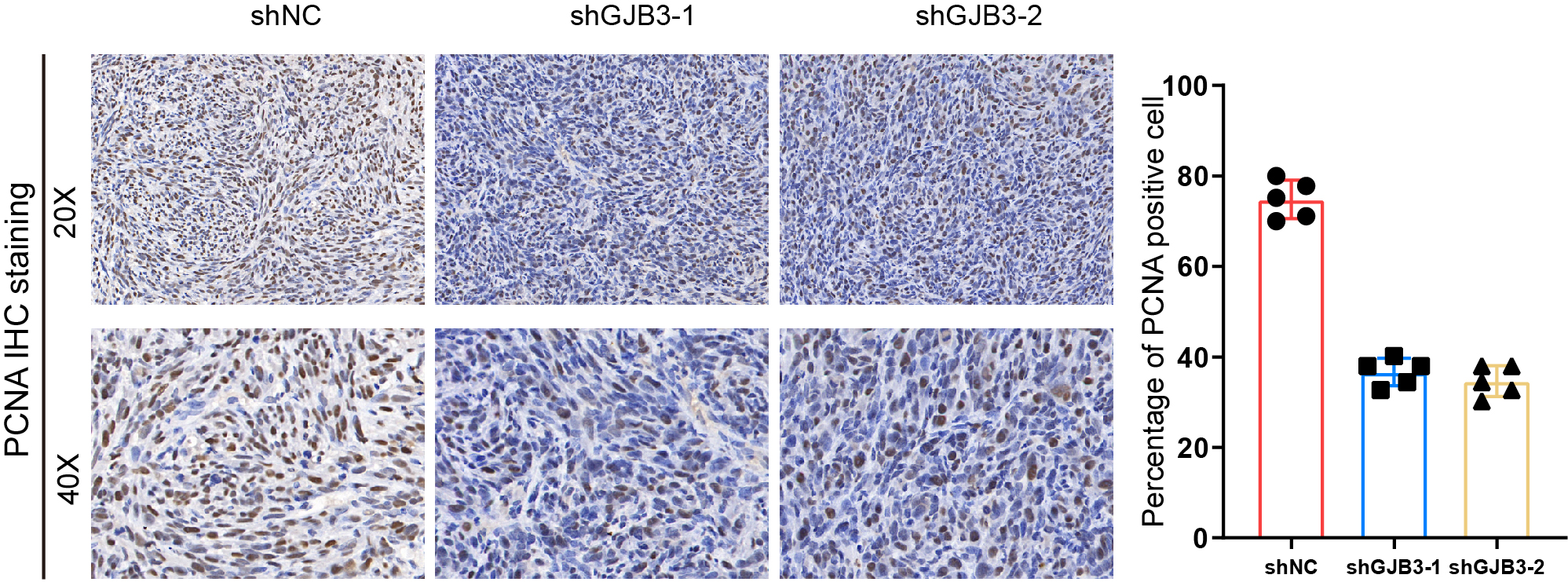


Supplementary figure 1 **GJB3 depletion reduced the progression of PDAC liver metastasis**

PCNA IHC staining of shNC and shGJB3 generated liver metastasis (left panel) and the statistic results of PCNA positive cells

Supplementary figure 2

GJB3


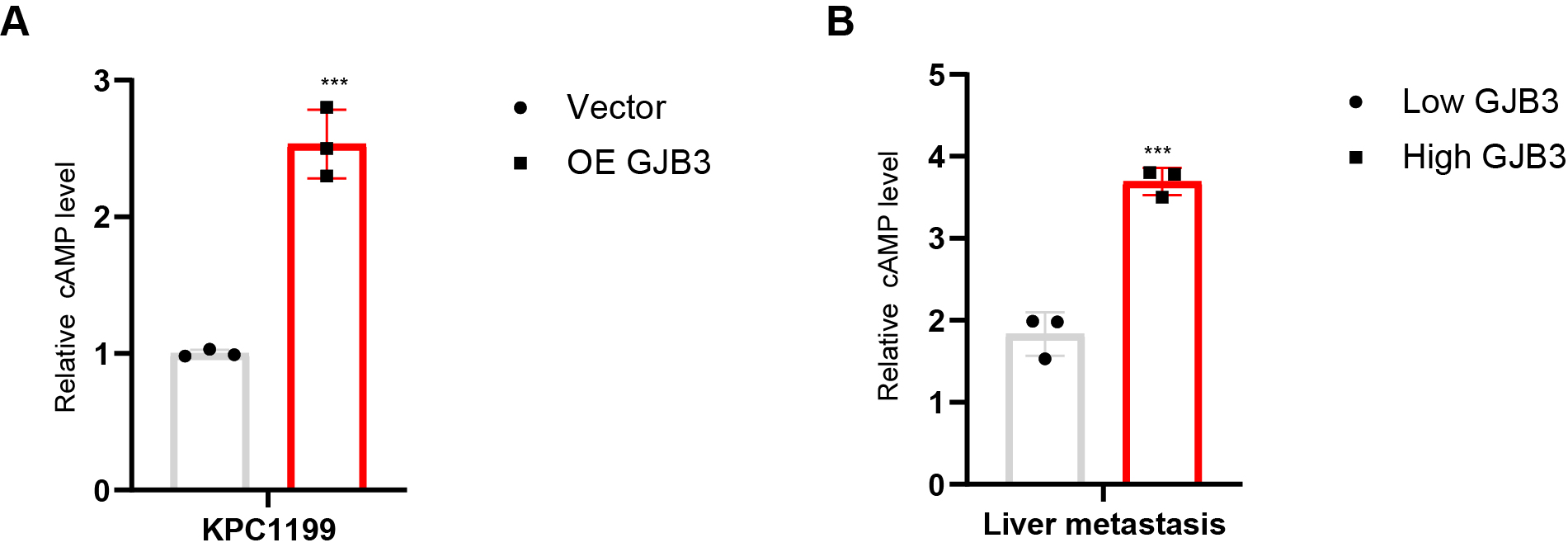


Supplementary figure 2 GJB3 enhance the cAMP transfer from tumor cells

A The quantification of cAMP in the culture supernatant from vector and OE GJB3 KPC1199 cells. B The quantification of cAMP in GJB3 high and low expression human liver metastasis specimens.
